# Resting-state functional MRI shows altered default-mode network functional connectivity in Duchenne muscular dystrophy patients

**DOI:** 10.1007/s11682-020-00422-3

**Published:** 2021-01-03

**Authors:** Nathalie Doorenweerd, Mischa de Rover, Chiara Marini-Bettolo, Kieren G. Hollingsworth, Erik H. Niks, Jos G. M. Hendriksen, Hermien E. Kan, Volker Straub

**Affiliations:** 1 John Walton Muscular Dystrophy Research Centre, Newcastle University and Newcastle Hospitals NHS Foundation Trust, Newcastle Upon Tyne, UK; 2 C.J. Gorter Center for High Field MRI, Department of Radiology, Leiden University Medical Center, C-03-Q, P.O. Box 9600, 2300 RC Leiden, The Netherlands; 3 Department of Anesthesiology, Leiden University Medical Center, Leiden, The Netherlands; 4 Clinical Psychology Unit, Institute of Psychology, Leiden University, Leiden, The Netherlands; 5 Newcastle Magnetic Resonance Centre, Translational and Clinical Research Institute, Faculty of Medical Sciences, Newcastle University, Newcastle upon Tyne, UK; 6 Department of Neurology, Leiden University Medical Center, Leiden, The Netherlands; 7 http://duchenneexpertisecentrum.nl; 8 Department of Neurological Learning Disabilities, Kempenhaeghe Epilepsy Center, Heeze, The Netherlands; 9 Department of Neurology, Maastricht University Medical Center, Maastricht, The Netherlands

**Keywords:** Duchenne muscular dystrophy, Resting state functional MRI, Default-mode Network, Attention/inhibition, Dystrophin

## Abstract

Duchenne muscular dystrophy (DMD) is an X-linked recessive neuromuscular disorder caused by absence of dystrophin protein. Dystrophin is expressed in muscle, but also in the brain. Difficulties with attention/inhibition, working memory and information processing are well described in DMD patients but their origin is poorly understood. The default mode network (DMN) is one of the networks involved in these processes. Therefore we aimed to assess DMN connectivity in DMD patients compared to matched controls, to better understand the cognitive profile in DMD. T1-weighted and resting state functional MRI scans were acquired from 33 DMD and 24 male age-matched controls at two clinical sites. Scans were analysed using FMRIB Software Library (FSL). Differences in the DMN were assessed using FSL RANDOMISE, with age as covariate and threshold-free cluster enhancement including multiple comparison correction. Post-hoc analyses were performed on the visual network, executive control network and fronto-parietal network with the same methods. In DMD patients, the level of connectivity was higher in areas within the control DMN (hyperconnectivity) and significant connectivity was found in areas outside the control DMN. No hypoconnectivity was found and no differences in the visual network, executive control network and fronto-parietal network. We showed differences both within and in areas outside the DMN in DMD. The specificity of our findings to the DMN can help provide a better understanding of the attention/inhibition, working memory and information processing difficulties in DMD.

**Supplementary Information:**

The online version contains supplementary material available at 10.1007/s11682-020-00422-3.

## Introduction

Duchenne muscular dystrophy (DMD) is an X-linked recessive neuromuscular disorder caused by mutations in the dystrophin encoding *DMD* gene. Dystrophin is expressed in numerous tissues in the body, including muscle and brain. Learning and behavioural problems in DMD are increasingly described Banihani et al. 2015; Hendriksen and Vles 2008; Ricotti et al. 2015a; Ricotti et al. 2015b), can occur both in patients with cognitive impairment as in those with IQs in the normal to high range (Battini et al. 2018), and can be detected early in development (Pane et al. 2013b). Specific problems include difficulty with verbal short term memory, visuospatial long term memory and verbal fluency Bresolin et al. 1994; Dorman et al. 1988; Pane et al. 2013b; Ricotti et al. 2015b). Increased incidence of neurodevelopmental disorders such as autism spectrum disorder (ASD, up to 21%), attention deficit-hyperactivity disorder (ADHD, up to 31%), obsessive-compulsive disorder (OCD, up to 5%), dyslexia and anxiety with respect to their general occurrence have also been consistently reported in DMD Hendriksen and Vles 2008; Pane et al. 2013a; Ricotti et al. 2015b; Snow et al. 2013) although with high variability in their respective prevalence. Despite the high incidence of these disorders, very little information is available regarding the prescription of psychopharmacology in DMD as also highlighted at the 249th ENMC workshop (www.enmc.org) (Lionarons et al. 2019). Next to findings on cognitive function and behaviour, neuroimaging modalities have demonstrated smaller total brain and grey matter volume, altered white matter microstructure and reduced cerebral blood flow (Doorenweerd et al. 2017a, 2014; Lee et al. 2002; Lv et al. 2011; Yoshioka et al. 1980).

In addition to full length dystrophin, dystrophin isoforms, Dp140, Dp71 and Dp40 are expressed in the brain (Doorenweerd et al. 2017b; Lidov 1996; Waite et al. 2012). Depending on the mutation location within the *DMD* gene, expression of one or multiple of these proteins will be affected. The function of dystrophin isoforms in the brain remains unclear. Hypotheses include an anchoring role for GABA_A_-receptors in neurons (Dp427) where absence of dystrophin leads to extra-synaptic dislocation of GABA_A_. A similar anchoring was proposed for aquaporin-4.1 in astrocytes (Dp71), where absence of dystrophin could disrupt blood-brain-barrier integrity (Waite et al. 2012). More recently, roles in early development and axonal guidance (Dp140) or in cerebral vasculature and pericytes (Dp71) were proposed but the effect of dystrophin absence have not yet been investigated (Doorenweerd et al. 2017b; Naidoo and Anthony 2019). This represents a major gap in knowledge in the DMD field.

A genotype-phenotype relationship has been suggested (Desguerre et al. 2009; Ricotti et al. 2015b), where poorer performance on specific cognitive tasks such as information processing was seen in patients missing Dp140 in addition to Dp427 (Doorenweerd et al. 2014). The same group of patients also had smaller grey matter volume. However, no direct relationship between reduced grey matter volume and cognitive function was found within genotyped subgroups, and the exact function of the brain dystrophin isoforms remains unclear.

One approach to try and bridge the gap between neuroimaging and cognition is the use of functional magnetic resonance imaging (fMRI) to assess the composition and strength of functional networks. A first step is the use of resting-state fMRI (RS-fMRI), which defines functional networks in absence of any specific cognitive tasks as regions with a synchronized oxygen uptake measured by a blood-oxygen-level dependent (BOLD) MRI sequence. Importantly in our cohort, it can be employed in patients irrespective of motor function or intellectual capacity (Damoiseaux et al. 2006; Smith et al. 2009). Of the ten functional networks that can be detected with RS-fMRI (Damoiseaux et al. 2006), the default mode network (DMN) is of particular interest in DMD as it is involved in functions such as working memory, retrieval of episodic memory, sustained attention and the interplay between emotional processing and cognitive functions (Mohan et al. 2016; Smith et al. 2009). The DMN is active in rest and suppressed during the performance of a specific cognitive task (Greicius et al. 2003). Structures generally included are the medial prefrontal cortex, the posterior cingulate cortex, the inferior parietal lobule, the lateral temporal cortex and the hippocampal formation (Andrews-Hanna et al. 2010).

Altered connectivity of the DMN has been reported for neurodevelopmental diseases such as ASD and ADHD Bos et al. 2017; Di Martino et al. 2014; Di Martino et al. 2017; Hahamy et al. 2015; Lee et al. 2016; Mohan et al. 2016; Silberstein et al. 2016). Compared to controls, alterations include hyper- and hypo-connectivity in different parts of the DMN based on the increase or decrease of the levels of synchronization. Besides changes in synchronization levels, patients also showed synchronous activity with additional brain regions compared to the control DMN.

There is currently only one report of functional connectivity in DMD patients. In that study, functional connectivity was investigated within the motor cortex of DMD patients, demonstrating decreased synchronization compared to controls that correlated to muscle function (Lv et al. 2011). However, this observation is difficult to relate to the cognitive phenotype of patients, as the progressive muscle wasting may well affect functional connectivity in the motor network.

As part of a larger ongoing study, containing longitudinal assessment of brain structure, function and metabolism we included RS-fMRI. Because the neuropsychological profile in DMD contains several elements that relate to the DMN in particular, we aimed to determine if there are differences in DMN connectivity between boys and young men with DMD compared to age-matched healthy males. This could constitute a biological correlate that might help explain part of the cognitive profile in DMD.

## Materials & methods

### Participants

This study took place in two sites, at LUMC, Leiden, The Netherlands and Newcastle University, Newcastle upon Tyne, in the United Kingdom, and was approved by the local medical ethic committees in both sites. Participants were recruited from patient registries, local clinics and flyers. Written informed consent was received from all participants and/or their legal guardians. Inclusion criteria for the DMD group were limited to a genetically confirmed diagnosis of DMD, being at least eight years of age and having the ability to lie supine for at least 30 minutes to obtain a random sample of the wide range of cognitive and genetic phenotypes. We did not stratify for corticosteroid regimes or other medication use. Healthy males were recruited through flyers at local schools and sports clubs and were age-matched to participants with DMD. MR contraindications were exclusion criteria for all candidates. Participant characteristics for the total cohort, and split per site, are shown in Table 1. The Brooke scale for upper extremity function and Vignos scale for lower extremity function (Brooke et al. 1987) were used as a gross classification of muscle function to indicate the stage of the disease. Based on the known mutations within the *DMD* gene, expression of one or more dystrophin proteins were predicted to be affected, dividing patients into those only missing Dp427, those missing Dp427 and Dp140, those missing Dp427, Dp140 and Dp71 and those where it was not possible to predict if expression of Dp140 would be impaired (supplementary material A contains a detailed description of the subdivision).

**Table 1:** Participant characteristics

	DMD			Control		
	Total	NL	UK	Total	NL	UK
General characteristics						
n=	**33**	21	12	**24**	17	7
Age mean, ± SD (years)	**13.4** ± **3.8**	12.8 ± 2.9	14.5 ± 5.0	**13.2** ± **2.6**	13.2 ± 2.0	13.3 ± 3.9
Age, range (years)	**8–21**	8–18	8–21	**8–20**	8–16	9–20
Dominant hand n=	**23 R / 10 L**	15 R / 6 L	8 R / 4 L	**21 R / 3 L**	15 R / 2 L	6 R / 1 L
Steroid treatment n=	**29**	17	12			
Deflazocort daily n=	**6**	1	5			
Prednisolone daily n=	**4**	0	4			
Prednisolone intermittent* n=	**18**	16	2			
Switched regimes n=	**1**	0	1			
Brooke scale, mean + SD	**2 ± 1.5**	2 ± 1.6	2 ± 1.2	**1**	1	1
Vignos scale, mean + SD	**6 ± 3.1**	6 ± 3.1	5 ± 3.0	**1**	1	1
Wheelchair bound n=	**15**	11	4			
Dystrophin proteins affected						
Dp427, n=	**12**	7	5			
Dp427 and Dp140, n=	**12**	11	1			
Dp427, Dp140 and Dp71, n=	**4**	0	4			
Dp427 and possibly Dp140, n=	**5**	3	2			

*Intermittent regimes include 1/1 day on/off, 7/7 days on/off and 10/10 days on/off

### Neuropsychological profile

In order to get an indication of the cognitive function of the cohort, a condensed neuropsychological examination (NPE) was administered. The test-battery was specifically compiled for this study to ascertain domains known to be frequently affected in DMD. The general intellectual level was assessed by the Peabody Picture Vocabulary test (PPVT-III-NL or PPVT-IV-ENG). This test measures receptive vocabulary, is normalised for age and requires no motor response. It was previously used in 130 DMD patients(Cyrulnik et al. 2007).

The reading composite score (standardized for age with range = 1–19, mean = 10, and standard deviation = 3 in healthy controls) was based on the monosyllabic word reading test and the 1-minute reading test derived from CB&WL (“continu benoemen en woorden lezen”; Bos & Lutje Spelberg, Boom test uitgevers, Amsterdam, the Netherlands) or the Test of Word Reading Efficiency, second edition (TOWRE-2, PRO-ED 2012) reading assessment. The information processing composite score (standardized for age with range = 1–19, mean = 10, and standard deviation = 3 in healthy controls) used 2 subtests from the Kaufman Assessment Battery (number recall for auditory working memory and block counting for conceptual thinking) and 1 subtest from the Wechsler Intelligence Scale for Children (symbol search). The third composite score for emotional and behavioural problems (range = 0–40) was constructed on the basis of the four problem-based subscales from the Dutch or English version of the Strengths and Difficulties Questionnaire for parents.

### MRI acquisition

Each site used a 3T scanner (Philips Achieva; Philips Healthcare, Best, the Netherlands) with an 8-channel receive-only head coil and used the same scan protocol. Participants were placed head-first supine with legs slightly elevated for comfort when desired. A parent or guardian could remain with them in the scanner room. Earplugs and headphones were used to dampen the noise of the scans and to communicate with and motivate the participants between scans. Music of the participants’ choice was played over the headphones during the anatomical scans. During the resting-state fMRI scan participants were instructed to keep their eyes open (without fixation) to prevent them from falling asleep and the music was switched off.

A three-dimensional T1-weighted scan (T1w; echo time (TE) = 4.6 ms, repetition time (TR) = 9.8 ms, spatial resolution (SR) = 1.17 × 0.92 × 1.2 mm, 140 slices, flip angle = 8 degrees, 4:55 minutes) was acquired for anatomical reference. A resting-state functional BOLD scan (RS-fMRI; FEEPI, TE/TR = 30 ms / 2200 ms, SR = 2.75 × 2.75 × 2.72 mm, 160 dynamics, 38 slices, flip angle = 80 degrees, EPI factor = 29, SPIR fat suppression, 6:01 min) was made to assess the functional connectivity.

### MRI data processing

FSL v.5.0.8 (Jenkinson et al. 2012) was used for all MRI data processing. On the T1w scans, brain extraction was performed using FSL BET2 (Smith 2002) with a threshold of 0.35 and additional clean-up of eyes and neck. Pre-processing was performed on the RS-fMRI data to determine the amount of head motion and remove any datasets containing obvious artefacts (i.e. field-of-view does not contain the entire cerebral cortex or signal voids at the frontal or temporal lobes) (Nickerson et al. 2017). Subsequently, the RS-fMRI data was prepared for ICA-based Automatic Removal Of Motion Artifacts (AROMA) (Pruim et al. 2015) using FSL FEAT (Woolrich et al. 2004) without temporal filtering, including 5 mm Full-Width Half-Maximum spatial smoothing and calculation of registration to the MNI152 template. ICA-AROMA, which uses Python v2.7 in addition to FSL, was then run on the FSL FEAT output, followed by temporal filtering and registration.

To have an age appropriate template, study specific networks best matching the ten standard resting-state networks (Smith et al. 2009) were obtained with ICA MELODIC (Beckmann et al. 2005) in the control group with 15 components with registration via the brain extracted T1w scans, a 2 mm resampling resolution, including components for noise and cerebrospinal fluid (CSF). A regression was then performed to obtain the same 15 networks in the DMD datasets. The DMN, visual network, executive control network and frontoparietal networks were selected from these 15 networks after visual inspection (supplementary material C contains the images of the networks). All 15 spatial maps from the group-average analysis were then used to generate participant-specific versions of the spatial maps, and associated time series, using dual regression (Nickerson et al. 2017). First, for each participant, the group-average set of spatial maps was regressed (as spatial regressors in a multiple regression) into the participant’s 4D space-time dataset. This resulted in a set of participant-specific time series, one per group-level spatial map. Next, those time series were regressed (as temporal regressors, again in a multiple regression) into the same 4D dataset, resulting in a set of participant-specific spatial maps, one per group-level spatial map.

### Statistics

FSL randomise (Winkler et al. 2014) was used to assess differences between DMD and controls for the DMN as the primary outcome, the visual network as negative control and the executive control network and frontoparietal networks as secondary outcomes. Age was taken along as a covariate and threshold-free cluster enhancement (TCFE) (Smith and Nichols 2009) including multiple comparison correction was used (p < 0.05). The group comparisons for the neuropsychological tests were made with T-tests with Bonferonni-Holmes (Benjamini et al. 2001) multiple comparison correction. Post-hoc testing was performed on the SDQ subscores, using T-tests without correcting for multiple comparisons. FSL randomise was finally used to post-hoc assess a potential effect of left versus right handedness on the significant RS-fMRI results.

### Data availability statement

Data available on request from the authors due to privacy/ethical restrictions.

## Results

A total of 37 males with DMD and 27 healthy age-matched males were recruited. Seven datasets contained artefacts as described in the methods (DMD n = 4, control n = 3) and were excluded from the analyses. The participant characteristics of the remaining 33 DMD and 24 controls are shown in Table 1. A subgroup analysis based on mutation group was not possible due to the limited number of patients in each group (n = 12, n = 12 and n = 4 respectively). This is therefore only reported as part of the cohort description. The RS-fMRI absolute mean displacement after motion correction was 0.03 mm (median = 0.03 mm, range = 0.01–0.07 mm).

**Fig. 1:**
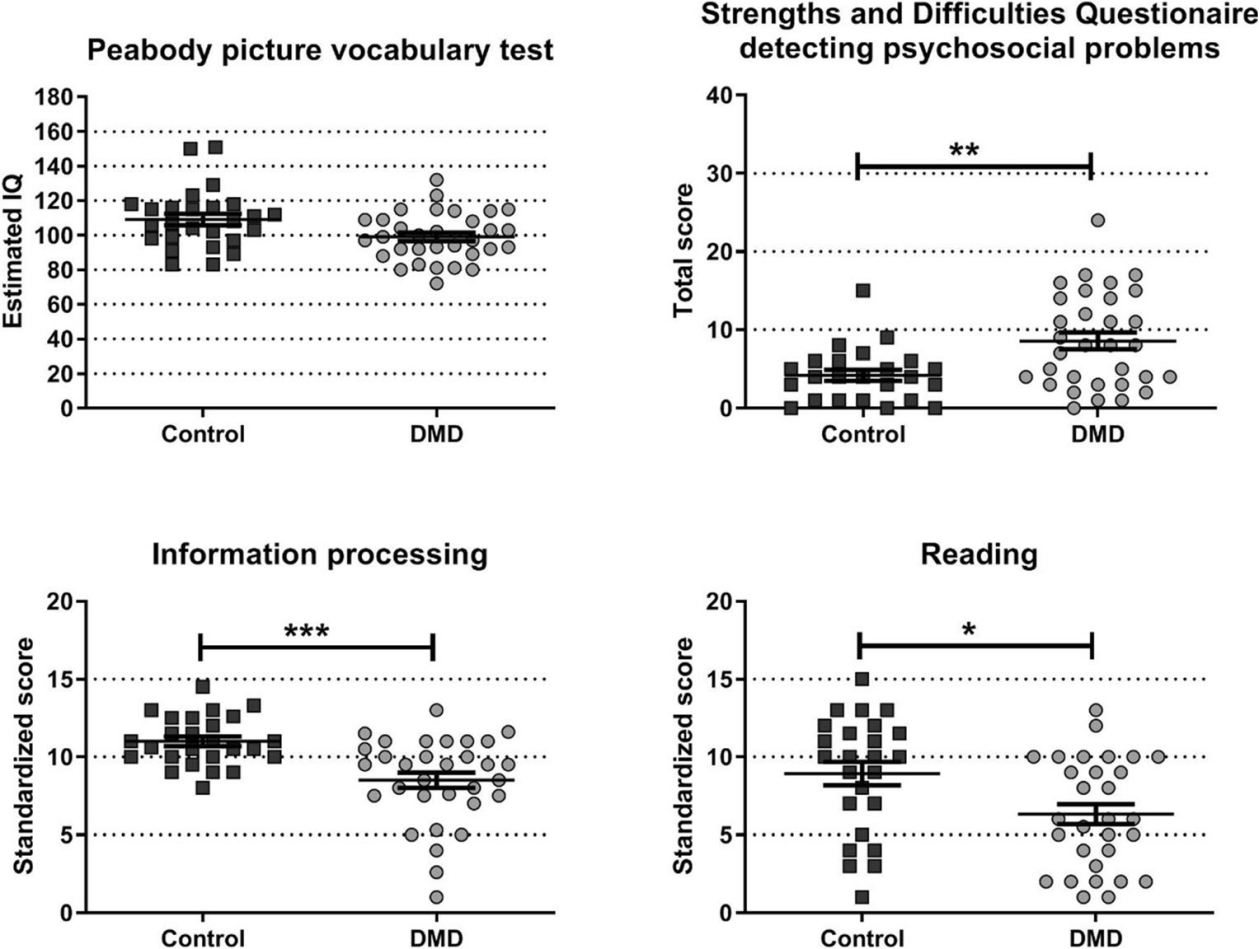
An indication of the cognitive function of the cohort with estimates of IQ, strengths and difficulty scores and composite scores for information processing and reading. * *p* < 0.05, ** *p* < 0.01, *** *p* < 0.001 after correcting for multiple comparisons

**Fig. 2:**
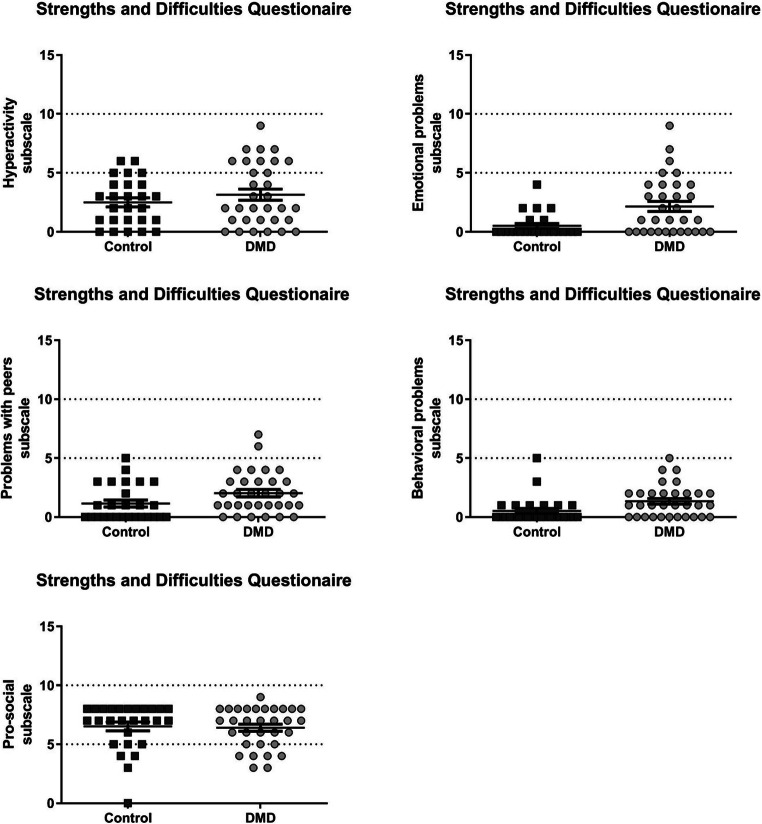
Scores on the subscales of the strengths and difficulties questionnaire. Post-hoc testing (uncorrected for multiple comparisons) showed higher scores on the hyperactivity, emotional problems, problems with peers and behavioural problems subscales which indicate potential difficulties and add up to the SDQ total score. Higher scores on the pro-social subscale indicate potential strengths

The scores of the Peabody picture vocabulary test, strengths and difficulties questionaire, information processing test, and reading test are shown in Fig. 1. Scores of the subcomponents of the strength and difficulties questionnaire can be found in Fig. 2. The groups did not differ with respect to their estimated general intellectual functioning. However, the DMD group had higher scores for psychosocial problems and lower scores for information processing and reading. Six of the control participants who had previously been diagnosed with dyslexia, showed the lowest scores on this composite score.

**Fig. 3:**
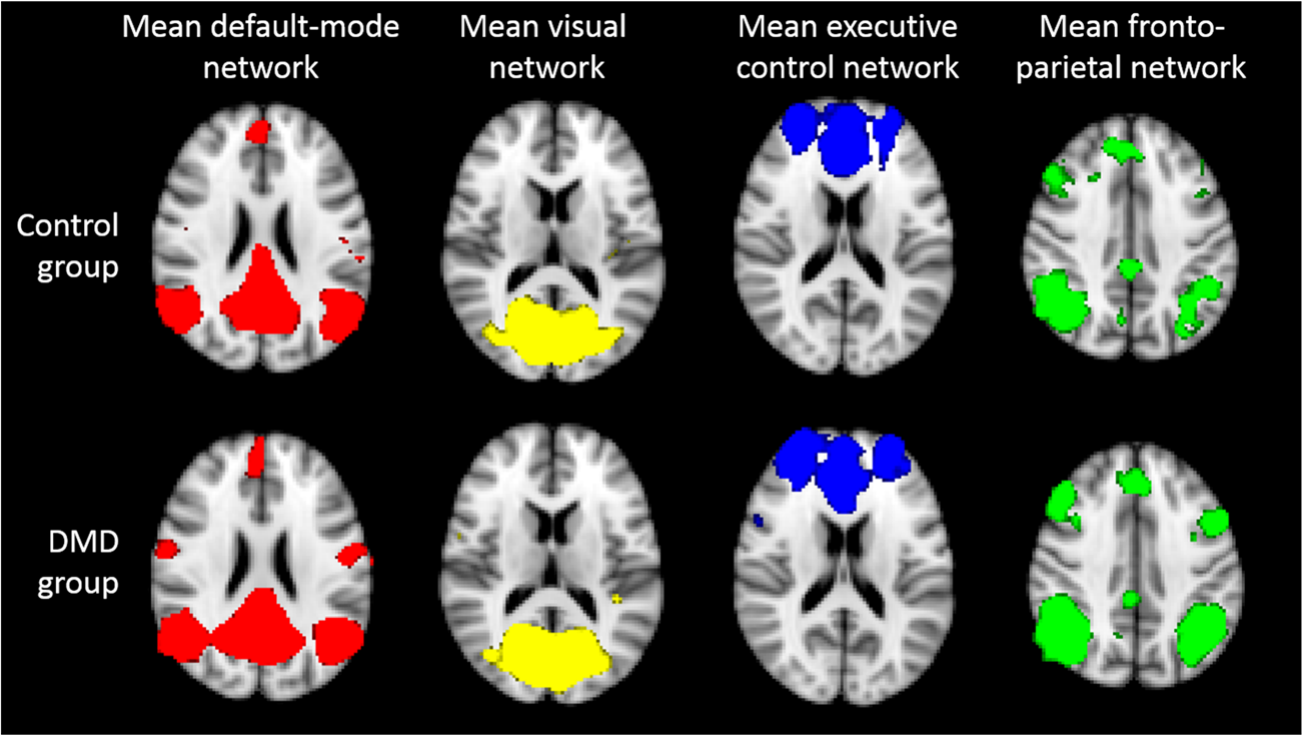
On a MNI standard background the mean networks are depicted separately for the control group (top row) and DMD group (bottom row). The default-mode network is shown in red, the visual network in yellow, the executive control network in blue and the fronto-parietal network in green

**Fig. 4:**
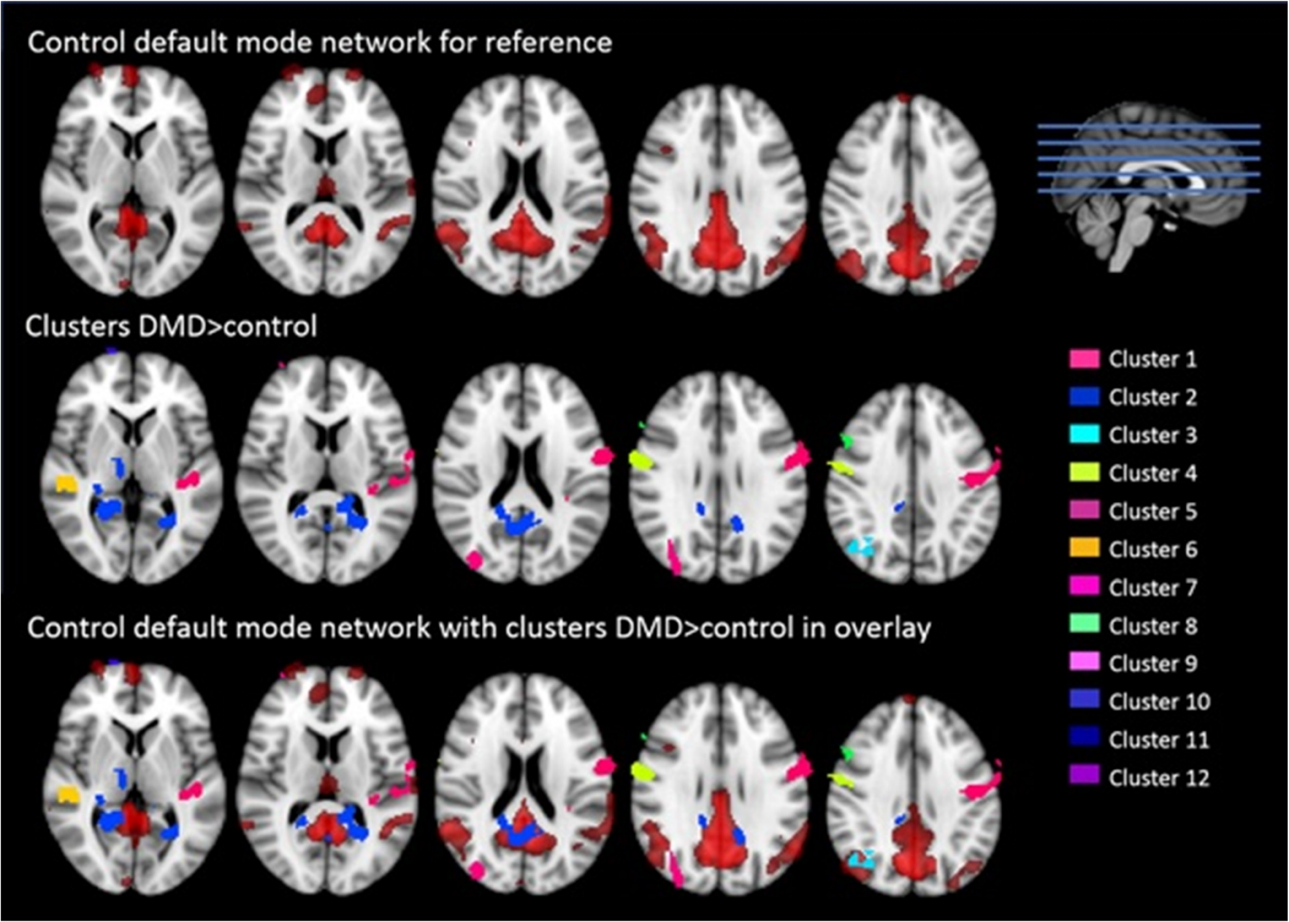
On a MNI standard background results of the group comparison in the default mode network are shown in five cross-sections. The control DMN is shown in red with significant clusters shown in all other colours. Only differences where DMD > control were found. Some cluster partially overlap with the DMN (for example cluster 2 or 3) and some extend the DMN (for example cluster 1, 4 or 6)

The group mean networks are depicted in Fig. 3 to demonstrate that each of the four networks could be detected separately in the control group and in the DMD group. No differences were found between DMD and controls within or in relation to the visual network, executive control network or frontoparietal network. In contrast, Fig. 4 represents the anatomical localisation of twelve significant anatomical clusters associated with the DMN. In DMD patients, the level of connectivity was higher in specific areas within the control DMN (hyperconnectivity) or a significant connectivity was found in areas outside the control DMN further referred to as additional connectivity. No hypoconnectivity was found in DMD patients. A full list of the 12 anatomical clusters that differed between DMD and controls shown in Table 2, ranked by size from largest to smallest and further described based on the Harvard-Oxford Brain Atlas (Desikan et al. 2006). The largest cluster was found in Heschl’s gyrus, projecting to the precentral and postcentral gyrus, in which also cluster four and cluster twelve were found. These are all part of the primary auditory cortex, the primary motor cortex and the primary somatosensory cortex. The precuneous cortex was part of the second and seventh clusters projecting to the lingual gyrus and the cingulate gyrus respectively. These areas are involved in episodic memory, visuospatial processing, and reflections upon self, i.e. classic examples of functions of the DMN. Part of the lateral occipital cortex, another component of the DMN in controls, was found in the third and fifth clusters. The superior temporal gyrus, which is involved in auditory processing including language comprehension, and is also important for social cognition, was found in the sixth cluster. The eighth cluster was found in the middle frontal gyrus projecting to precentral and inferior frontal gyrus. These brain regions are involved in language processing and speech production. The frontal pole was identified in clusters nine and ten, which is suggested to be involved in memory recall. Finally, the right pallidum projecting to right putamen, involved in the regulation of voluntary movement, was identified for cluster eleven.

**Table 2:** List of clusters (all DMD > control) with annotations

Cluster	Size (voxels)	Voxels max	Coordinates max (x/y/z)	Annotation (Harvard-Oxford Cortical Structural Atlas)
1	1529	4.31	-48/-24/8	Heschl’s gyrus (includes H1 and H2) projecting to precentral and postcentral gryus
2	1409	4.7	-24/-58/6	Precuneous cortex projecting to lingual gyrus
3	389	4.56	34/-68/40	Lateral occipital cortex, superior division
4	268	4.24	58/-2/32	Precentral gyrus projecting to postcentral gyrus
5	227	3.87	34/-84/24	Lateral occipital cortex, superior division projecting to occipital pole
6	179	4.62	50/-26/2	Superior temporal gyrus, posterior division
7	84	3.74	10/-52/50	Precuneus cortex projecting to cingulate gyrus
8	52	3.71	48/18/34	Middle frontal gyrus projecting to precentral and inferior frontal gyrus
9	19	3.9	34/64/10	Frontal pole
10	14	4.46	16/74/4	Frontal pole
11	13	4.1	16/74/4	Right pallidum projecting to right putamen
12	10	3.11	26/-8/-6	Precentral gyrus projecting to postcentral gyrus

There was no effect of left or right handedness on the DMN results. With respect to a potential effect of age on the results, only a significant positive correlation with age was found in the visual network which did not lead to a difference in the between group results.

## Discussion

The aim of the current study was to compare DMN connectivity between males with DMD and age- and sex-matched controls, to determine if there is a neurobiological correlate that might help explain difficulties with attention/inhibition, working memory and information processing in this condition. In DMD patients, we found significant hyper-connectivity within the control DMN and significant connectivity in areas outside the control DMN (additional connectivity).

In agreement with the literature, the DMN in controls contained the posterior cingulate and adjacent precuneus, the bilateral inferior-lateral-parietal, and the ventromedial frontal cortex. (Smith et al. 2009) A midline core is usually defined within the DMN by (regions within) the posterior cingulate and anterior medial prefrontal cortex that is active when people make self-relevant, affective decisions. Another part of the DMN is the medial temporal lobe subsystem which is engaged when decisions involve constructing a mental image based on a memory. (Andrews-Hanna et al. 2010) Overall, the DMN is involved in retrieval of episodic memory (personal memories, personal facts, nonpersonal memories, nonpersonal facts) and working memory. (Maguire and Mummery 1999) Rather than affecting only a sub-part of the DMN, the differences observed in DMD were spread throughout the DMN, encompassing all functions mentioned above. The additional connectivity with the primary motor cortex and primary somatosensory cortex may well be related to the fact that DMD is a neuromuscular disorder. The connectivity with the primary auditory cortex, lingual gyrus, superior temporal gyrus and middle frontal gyrus may also reflect altered language comprehension. The auditory input from noise exposure inherent to MRI was equal for all participants.

The neuropsychological profile of our cohort was similar to that reported in the literature, with mildly reduced mean IQ, higher problem scores on the SDQ, and higher incidence of difficulties with information processing and reading. It would be interesting to determine if there is a direct correlation between these scores and the DMN connectivity strength within the DMD group. Unfortunately, the n = 33 DMD fMRI datasets do not provide sufficient statistical power for reliable correlation analyses with the NPE scores.”

The DMN has an inverse correlation with task-related activity. When a task is being performed the DMN is deactivated and when no task is being performed the DMN becomes active. (Greicius et al. 2003) Hyper-connectivity within the DMN may result in stronger deactivation of the DMN when a task is performed. This has been demonstrated in patients with ADHD. Stronger functional connectivity was also found in the DMN in ADHD and the inverse relationship between the DMN and the cognitive control network was attenuated in ADHD. (Mohan et al. 2016) Patients with ADHD showed reduced inhibition of the DMN while performing a cognitive task which could be influenced by treatment with methylphenidate, which supresses the increased functional connectivity of the DMN and was associated with improved performance. (Silberstein et al. 2016) The comorbidity of ADHD in DMD may be related to the alterations to the DMN. Attenuation of the cognitive control network was also found in ADHD. Due to time constraints and burden limitation, we performed an abbreviated neuropsychological assessment, which prevented assessment of validated information on comorbidities within our cohort. However, because our results in the DMN were largely similar to findings in ADHD, we also checked the executive control network and frontoparietal network as the closest match to the cognitive control network. We did not find any significant differences in either network, distinguishing our results in DMD from those found in ADHD.

Alterations to the DMN have also been reported for ASD, which also has a high comorbidity in DMD. In ASD, both hyper- and hypo-connectivity have been reported in the DMN, but hypo-connectivity dominated.(Di Martino et al. 2014; Lynch et al. 2013; Monk et al. 2009) Distorted topographical patterns were also seen. (Hahamy et al. 2015) It is suggested that this heterogeneous profile relates to maturation abnormalities, yet some aspects of the neural signatures of ASD remain constant throughout brain development. (Di Martino et al. 2014) It would be of interest to investigate maturation further in DMD. One of the brain dystrophins, Dp140, is expressed mostly during fetal development. (Doorenweerd et al. 2017b) It remains unclear whether the cognitive phenotype reflect progressive deterioration or developmental delay. Based on the findings in ASD, the altered topographical pattern in the DMD DFN would point in the direction of developmental delay. A longitudinal study design would be required to address this question.

It is unknown what effect the use of corticosteroids may have on functional activity of the brain as detected by RS-fMRI BOLD. As the vast majority of patients was taking corticosteroids, with different regimes, reflecting current standard of care, we were not able to explore this potential confounding effect. Corticosteroids can have side effects that may also influence mood or behaviour. (Hendriksen et al. 2017; McDonald et al. 2018; Sienko et al. 2016) Ongoing trials are investigating potential alternatives for corticosteroids, which could open up the possibility to assess to what extend our findings are a consequence of the corticosteroids in the future (clinicaltrials.gov/ct2/show/NCT03439670). Another possible confounding effect in the data was the reduced cerebral perfusion in DMD (Doorenweerd et al. 2016) which could impact the signal-to-noise ratio. We therefore assessed the visual network as negative control. If the differences found were due to a discrepancy in signal-to-noise ratio, we should have also found differences is the visual network, which we did not. Unfortunately, the patient subgroups based on dystrophin isoform expression were too small to be analysed separately. As such we are unable to determine if there is a genotype-phenotype association. Finally, it would be of interest to determine patient subpopulations based on full neuropsychological diagnostic evaluations. This could enable a more direct comparison to literature in ASD or ADHD.

Now that we have established that the DMN requires further investigation in DMD, we can direct future research where RS-fMRI can be combined with task-based fMRI and cognitive and neurobehavioral assessments with specific tasks relating to the default mode network to assess how they tie together.”

In conclusion, we have found altered DMN connectivity in DMD patients compared to controls. The hyper-connectivity within the DMN may result in greater deactivation when a task is performed due to the inverse relationship with task-related activity, similar to ADHD. The additional regions with the same activity patterns as the DMN may be indicative of alterations in maturation, similar to ASD. The specificity of our findings to the DMN and not the visual, executive control of fronto-parietal networks can help guide basic research to this specific brain network. Overall, our findings can provide a better understanding of the attention/inhibition, working memory and information processing difficulties in DMD if explored further in a longitudinal format with task-based fMRI included and with full neuropsychological diagnostic classifications.

## Electronic supplementary material

ESM 1 (DOCX 1.27 MB)
