# Supplementary material for: Resting-state functional MRI shows altered default-mode network functional connectivity in Duchenne muscular dystrophy patients
Source: Brain Imaging Behav. 2021 Jan 3;15(5):2297–307. doi: 10.1007/s11682-020-00422-3 (PMC8500880; doi:10.1007/s11682-020-00422-3)
Supplement: Supplementary file 1 — (DOCX 1.27 MB) [file 11682_2020_422_MOESM1_ESM.docx]

**Supplementary material**


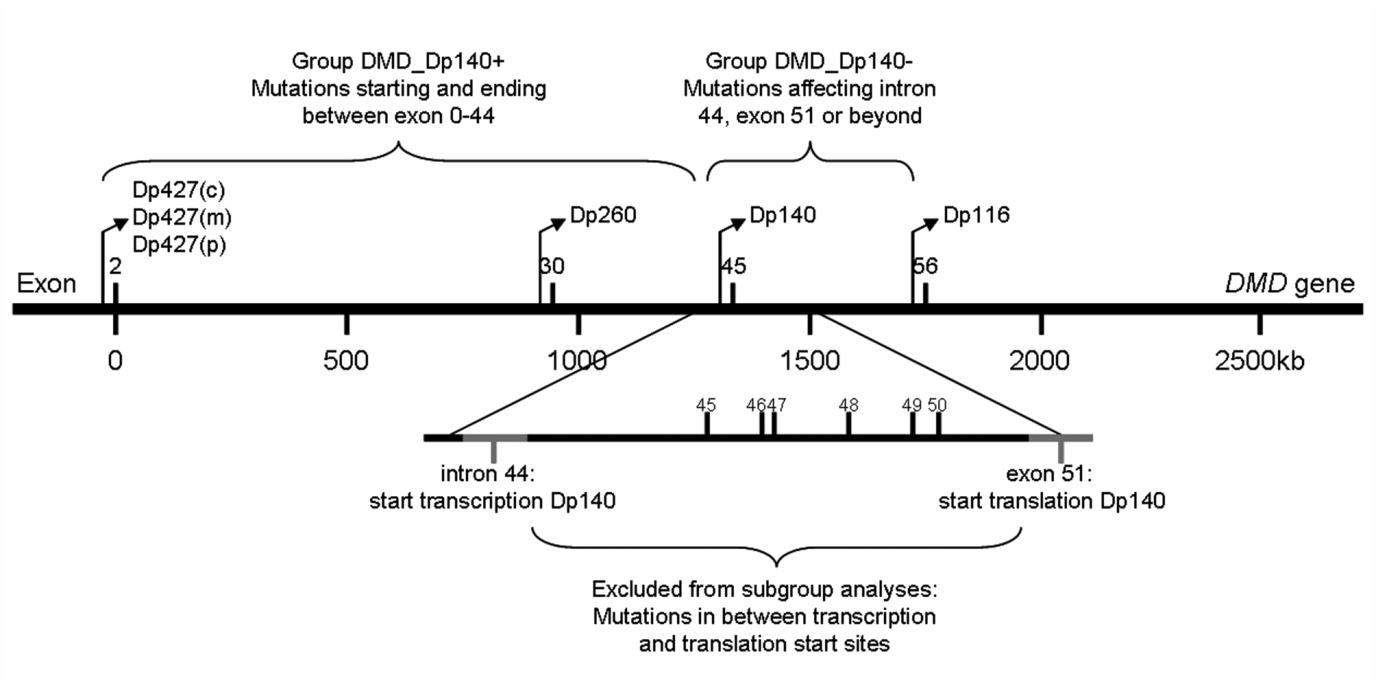


*Supplementary figure A: A schematic representation of the DMD gene and the location of the promotors for dystrophin. Dp427 contains exon 1-79. Dp140 start at intron 44 with transcription, but translation starts in exon 51 after which it contains all the remaining exons to 79. It is unknown what happens to the formation of Dp140 when the mutation begins and ends exactly in between the start of transcription and translation of Dp140. Dp71 starts at intron 62 and runs through to exon 79. Dp40 shares a start site with Dp71, but misses the C-terminal domain.*

*The group division was made as follows:*

- *Patients missing only Dp427 have a mutation that starts and ends in between exon 1 and 44.*
- *Patients missing Dp427 and Dp140 have a mutation that includes the transcription start site in intron 44, or includes exon 51 to 62.*
- *Patients with a mutation starting and ending in between exon 45 and 51 were excluded from the subdivision.*
- *Patients with a mutation that includes the transcription start site in intron 62 or exon 63-79 miss Dp427, Dp140 and Dp71. In our case the mutations did not affect exon 70-79 so patients in this group also miss Dp40* which has a stop codon before exon 70*.*

*
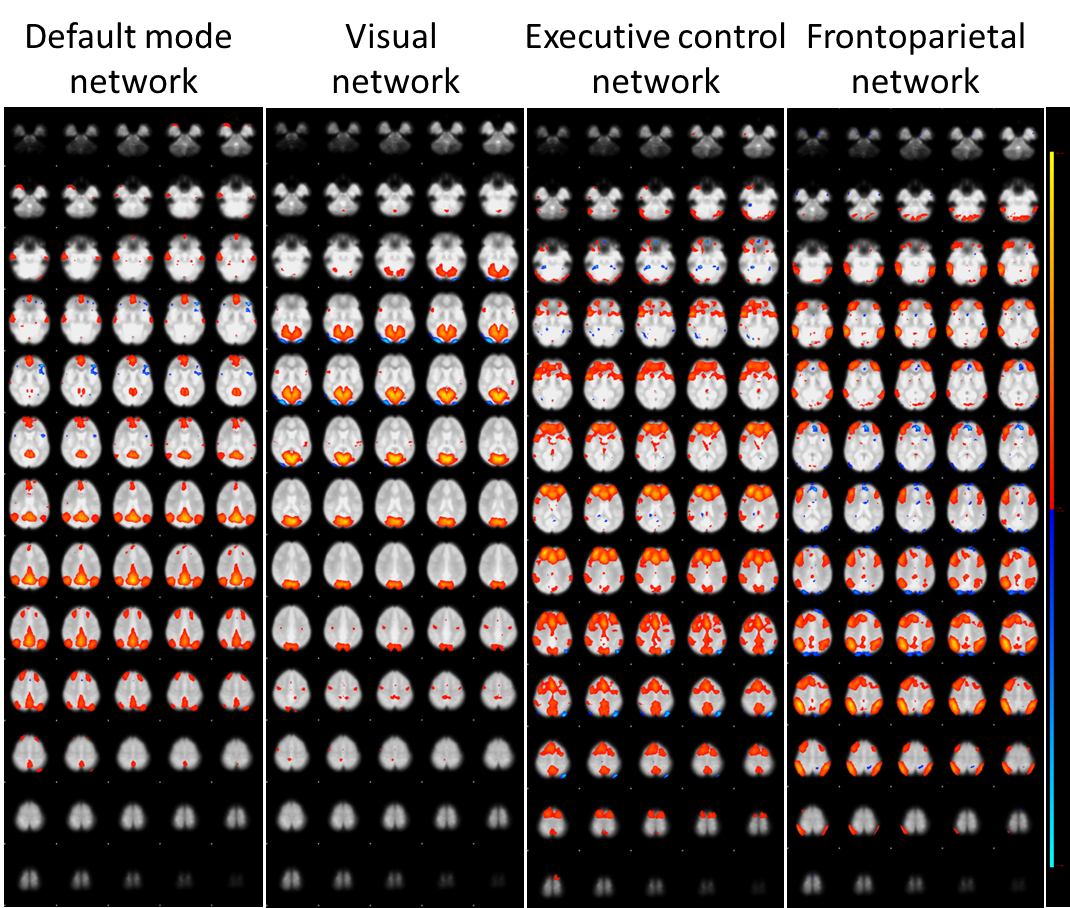
*

*Supplementary figure B: Networks that were identified in the healthy controls and that were used for the comparison with DMD*
